# Influence of Digital Workflows on Maxillary Complete Denture Base Fit Accuracy: A Prospective Clinical 3D Evaluation

**DOI:** 10.3390/jfb17090470

**Published:** 2026-09-15

**Authors:** Helin Su Akyol-Kurtca, Deger Ongul, Bilge Gokcen-Rohlig

**Affiliations:** 1 Department of Prosthodontics, Institute of Graduate Studies in Health Sciences, Istanbul University, 34452 Istanbul, Turkey; 2 Department of Prosthodontics, Faculty of Dentistry, Istanbul University, 34452 Istanbul, Turkey

**Keywords:** denture bases, 3D printing, digital denture, fit accuracy, intraoral scanning

## Abstract

Background: This study investigated the fit accuracy of maxillary complete denture bases (CDBs) fabricated using conventional, fully digital, and two hybrid workflows. Methods: Thirteen edentulous patients each received four maxillary CDBs produced via conventional (C), fully digital (FD), hybrid early (HE), and hybrid late (HL) workflows. Group C employed functional impressions and heat-polymerized polymethyl methacrylate (PMMA). Group FD utilized intraoral scanning (IOS), computer-aided design (CAD), and three-dimensional printing. The HE workflow combined digitally designed custom trays with conventional impressions, while the HL workflow digitized conventional master casts prior to digital fabrication. The master cast from the C workflow served as the reference. Deviations were calculated at 22 landmarks across six anatomical regions, with overall and regional analyses performed. Results: Group C exhibited the lowest deviation, followed by HE. HL and FD groups demonstrated significantly higher deviations (*p* < 0.001), with no significant difference between them. Regional variations were observed across most anatomical areas, except for the posterior palatal seal (*p* = 0.287). Conclusions: Clinical workflow significantly influences denture base fit accuracy. Conventional and hybrid early workflows provided more predictable adaptation, whereas hybrid late and fully digital workflows showed greater deviation, particularly in functional regions, potentially affecting clinical performance.

## 1. Introduction

Complete edentulism remains a widespread oral health issue, particularly among older adults, and it significantly impairs oral health-related quality of life by affecting essential functions and esthetics [1,2]. Complete dentures (CDs) fabricated using conventional techniques remain the most common rehabilitation method because of their cost-effectiveness, non-invasive nature, and broad applicability for patients with systemic or financial limitations [3,4]. Although still the clinical standard, conventional CD fabrication workflows require multiple patient visits, involve operator-dependent steps, and are prone to errors such as polymerization shrinkage, which may compromise fit, retention, and long-term patient comfort [5,6,7].

In 1994, Maeda et al. [8] introduced one of the first computer-aided design and computer-aided manufacturing (CAD/CAM) systems for CDs. These digital systems have been integrated into the CD fabrication workflow, offering potential advantages such as reduced chairside time, fewer laboratory steps, enhanced reproducibility, and digital data storage [9,10]. Digital workflows include three sequential stages: data acquisition, computer-aided design, and manufacturing via subtractive (milling) or additive (3D printing) processes [11,12]. Intraoral scanning has gained increasing attention in the data-acquisition stage and has advanced digital workflows by eliminating the need for conventional impression materials and improving patient comfort [13,14]. The use of intraoral scanners offers reliable accuracy for fixed prostheses; however, when applied to completely edentulous arches, it faces challenges in accurately capturing mobile and compressible soft tissues, particularly in functionally critical regions. In addition to intraoral scanners that directly digitize the edentulous arches, indirect methods allow for denture fabrication by digitizing either the functional impression or the master cast using an extraoral scanner [4]. Hybrid approaches extend these indirect methods by combining selected conventional procedures with digital design and manufacturing to maintain accuracy in functionally critical regions. Consequently, such combined protocols have been adopted to enhance fit accuracy in complete denture fabrication [15,16,17]. Fit accuracy of CDBs has increasingly been evaluated using three-dimensional (3D) surface superimposition techniques, enabling quantitative assessment of overall and region-specific deviations [7,9,18].

Complete denture bases can be fabricated digitally using either subtractive milling or additive 3D-printing techniques. Milling employs pre-polymerized PMMA blocks, thereby eliminating polymerization shrinkage and minimizing residual monomer release [11,19]. In contrast, 3D-printing techniques fabricate denture bases through sequential polymerization of photopolymer resins, enabling the fabrication of complex geometries with reduced material consumption. However, their accuracy and long-term stability may be influenced by resin properties, printing parameters, and post-processing protocols [9,20,21]. Regarding 3D printing, studies are heterogeneous: while some investigations reported comparable or superior outcomes compared with conventional methods [9,20], others reported less satisfactory results, highlighting the technique-sensitive nature of this approach [17,19].

Despite the expanding literature on digital complete denture fabrication, clinical evidence directly comparing fit accuracy across different workflows remains limited, particularly in anatomically and functionally critical regions. Therefore, this study aimed to evaluate and compare the overall and region-specific fit accuracy of complete denture bases fabricated using four clinical workflows. Two null hypotheses were tested: (1) overall fit accuracy would not differ among the workflows; (2) region-specific fit accuracy would not differ among the workflows.

## 2. Materials and Methods

This in vivo study was designed as a prospective, randomized, controlled clinical study with examiner-blinded outcome assessment and was conducted at the Department of Prosthodontics, Faculty of Dentistry, Istanbul University, between 12 March 2024, and 17 March 2025, in accordance with the ethical principles of the Declaration of Helsinki (2013 revision). Ethical approval was obtained from the Institutional Review Board of Istanbul University (No. 702; approval date: 27 February 2024), and written informed consent was obtained from all participants before enrollment. Because local regulations required only ethics approval at study initiation, ClinicalTrials.gov registration was completed retrospectively. The study has since been registered at ClinicalTrials.gov (identifier: NCT07795983).

**Figure 1:**
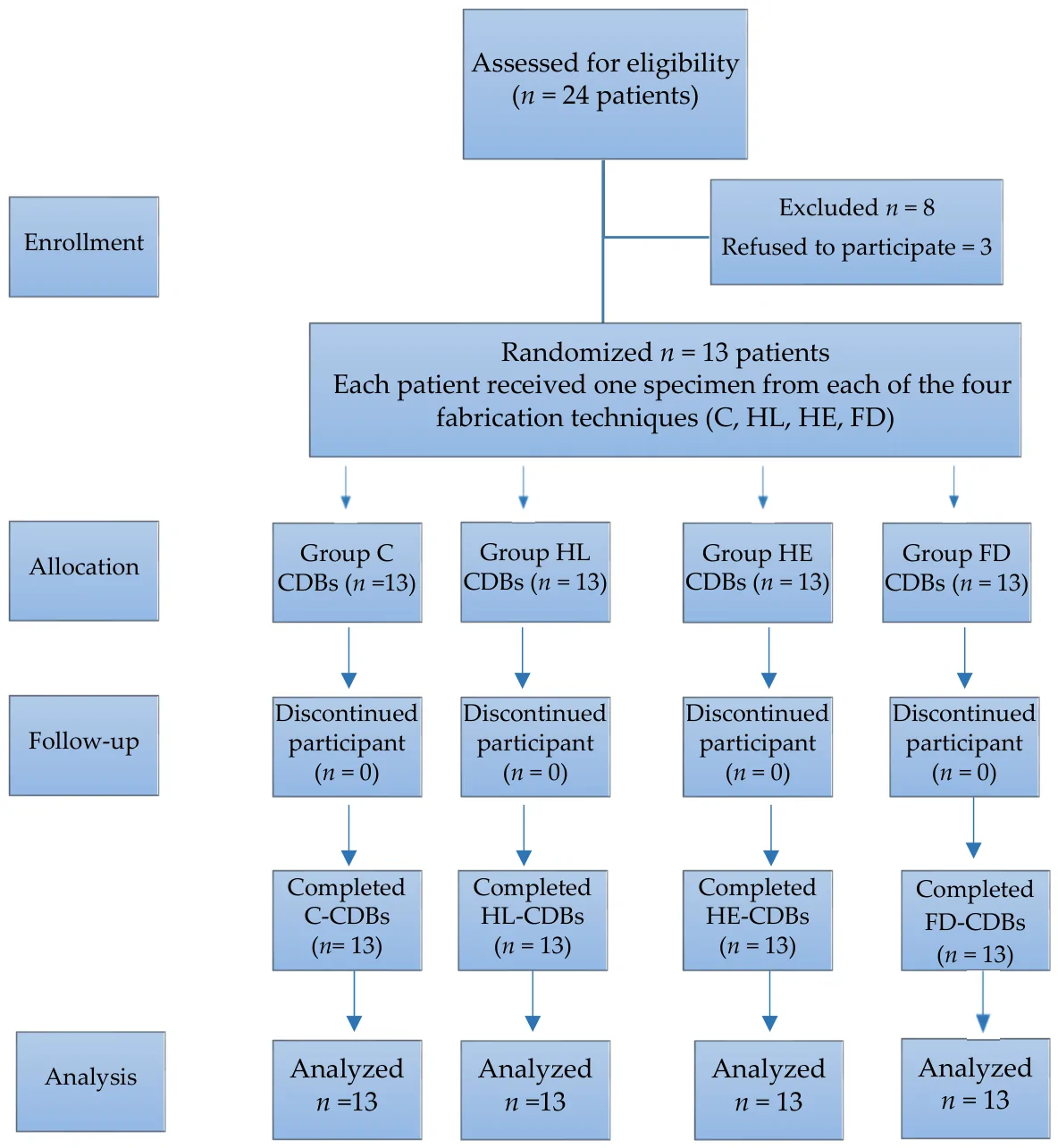
CONSORT flow diagram of participant enrollment, allocation, follow-up, and analysis.

An a priori power analysis was performed to determine the required sample size [22]. Using IBM SPSS Statistics Version 29 (IBM Corp., Armonk, NY, USA), a minimum of 10 patients was calculated to achieve 95% power at a 5% significance level. To compensate for potential dropouts, 13 participants were planned for recruitment. Patients scheduled for complete denture treatment at the Department of Prosthodontics were randomly selected from the waiting list. Those who met the inclusion criteria and provided written informed consent were enrolled in the study until the target sample size was reached. A total of 13 patients (58 ± 3 years; 7 males, 6 females) were included. Although participant sex was recorded as part of baseline characteristics, sex and gender were not analyzed as study variables because they were not relevant to the aims of this research. This randomized controlled trial was reported in accordance with the CONSORT reporting guidelines. The participant enrollment, allocation, follow-up, and analysis are presented in the CONSORT flow diagram (Figure 1).

**Figure 2:**
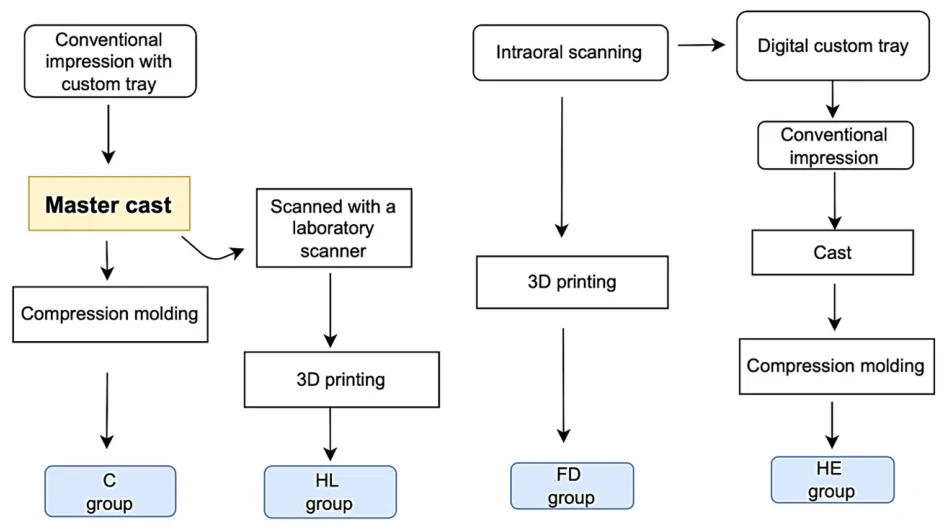
Workflow diagram of the study showing the fabrication procedures for each clinical workflow. C, conventional; FD, fully digital; HE, hybrid early; HL, hybrid late.

The inclusion criteria were being completely edentulous in the maxillary arch, age between 18 and 65 years, and having no systemic disease, psychiatric disorder, or a history of alcohol or drug abuse. Patients were excluded if they exhibited severe ridge resorption, extensive maxillary undercuts, dementia, malignant neoplasms, dependence on caregivers, or other conditions that could compromise treatment reliability and repeated clinical visits. For each patient, all fabrication workflows were performed, and four different maxillary CDBs were fabricated and assigned to four groups: conventional (C), fully digital (FD), hybrid early (HE), and hybrid late (HL) (Figure 2).

All conventional and digital clinical procedures related to denture base fabrication were performed by a single experienced operator to ensure procedural standardization across all workflows. Prior to patient enrollment, the operator underwent dedicated training and calibration in intraoral scanning, including multiple trial scans on edentulous patients, to familiarize themselves with the scanning protocol and minimize operator-related variability.

### 2.1. Fabrication of C Group CDBs

Group C served as the control group, and CDBs were fabricated using a conventional technique. At the first appointment, preliminary impressions were obtained using prefabricated edentulous trays (Dentaurum GmbH & Co. KG, Ispringen, Germany) and an irreversible hydrocolloid impression material (IQ Chrom, LASCOD, Florence, Italy). The impressions were poured with Type III dental stone (Extra Hard Dental Plaster, Cerestone, Turkey) to fabricate casts for custom tray fabrication.

Custom trays were fabricated in the laboratory by a single dental laboratory technician using a light-polymerized acrylic resin (Triad^®^ VLC System, Dentsply Sirona, Charlotte, NC, USA). The custom trays were trimmed approximately 2 mm short of the vestibular sulcus for border molding. After intraoral adjustment, the border extensions of the custom trays were evaluated using Herbst’s functional tests to verify their appropriate extension. Border molding was subsequently performed using a thermoplastic impression compound (Impression Compound, Kerr Corporation, Orange, CA, USA) to record functional muscle movements and anatomical extensions, including the posterior palatal seal.

Definitive functional impressions were then obtained using zinc oxide–eugenol impression paste (SS White, SS White Group, c/o Prima Dental Group, Gloucester, UK) and poured with Type IV dental stone (Elite Rock, Zhermack S.p.A., Badia Polesine, Rovigo, Italy) to obtain definitive casts, which served as the master casts. Complete denture bases were fabricated on the master casts using the conventional compression molding technique and heat-cured PMMA resin with a uniform denture base thickness of 2 mm (ProBase^®^ Hot, Ivoclar Vivadent, Sydney, Australia).

### 2.2. Fabrication of FD Group CDBs

The fabrication of CDBs in this group was performed using a fully digital workflow. The edentulous maxillary arches were scanned using an intraoral scanner (TRIOS 5, 3Shape A/S, Copenhagen, Denmark). Intraoral scanning followed the recommended protocol, starting from the maxillary tuberosity area and progressing along the residual ridge to the contralateral side, followed by palatal and buccal surface scanning. All scans were performed by a single calibrated operator, with the device calibrated prior to each scanning session. Each maxillary arch was captured in a single continuous scan (~45–70 s), and incomplete areas were rescanned until complete surface coverage was achieved.

The resulting STL files were transferred to the dental laboratory, and denture bases were digitally designed using CAD software (exocad GmbH, DentalCAD 3.0 Galway, Darmstadt, Germany). During the CAD process, denture base thickness was standardized at 2.0 mm. The datasets were then exported as STL files, and CDBs were fabricated using a liquid PMMA denture resin (PowerResins Denture Resin, 3BFAB, Istanbul, Turkey) with a DLP 3D printer (Sega DLP, DentaFab/3BFAB, Istanbul, Turkey).

After printing, the denture bases were removed from the build platform and cleaned according to the manufacturer’s protocol. The printed bases were rinsed in 99% isopropyl alcohol with agitation and brushing and then ultrasonically cleaned in alcohol for 10 min and air-dried. Subsequently, all bases were post-cured for 15 min in a dedicated UV polymerization unit (MEDIFIVE, Seoul, South Korea). Printing parameters followed standard denture base recommendations, including a 45° build angle, 50 µm layer thickness, and support placement away from the intaglio surface.

### 2.3. Fabrication of HE Group CDBs

The IOS datasets obtained from the FD group were used to design custom trays for the HE workflow. These trays were fabricated in the laboratory using a 3D printer (Phrozen Sonic Mini 8KS, Phrozen, Hsinchu, Taiwan). Following intraoral adjustment, the border extensions of the custom trays were evaluated using Herbst’s functional tests, and border molding and definitive functional impressions were performed according to the same protocol described for the C group. The definitive impressions were poured, and CDBs were fabricated on the resulting casts using conventional compression molding with heat-cured PMMA resin and a uniform denture base thickness of 2 mm.

### 2.4. Fabrication of HL Group CDBs

This group combined conventional impression procedures with digital fabrication. The master casts obtained in the C group were scanned in the laboratory using a desktop scanner (Shining 3D DS-EX, Shining 3D, Hangzhou, China). The resulting STL files were imported into CAD software for denture base design, and CDBs were subsequently fabricated using the same 3D-printing workflow and post-processing protocol described for the FD group.

### 2.5. Evaluation Parameters

**Figure 3:**
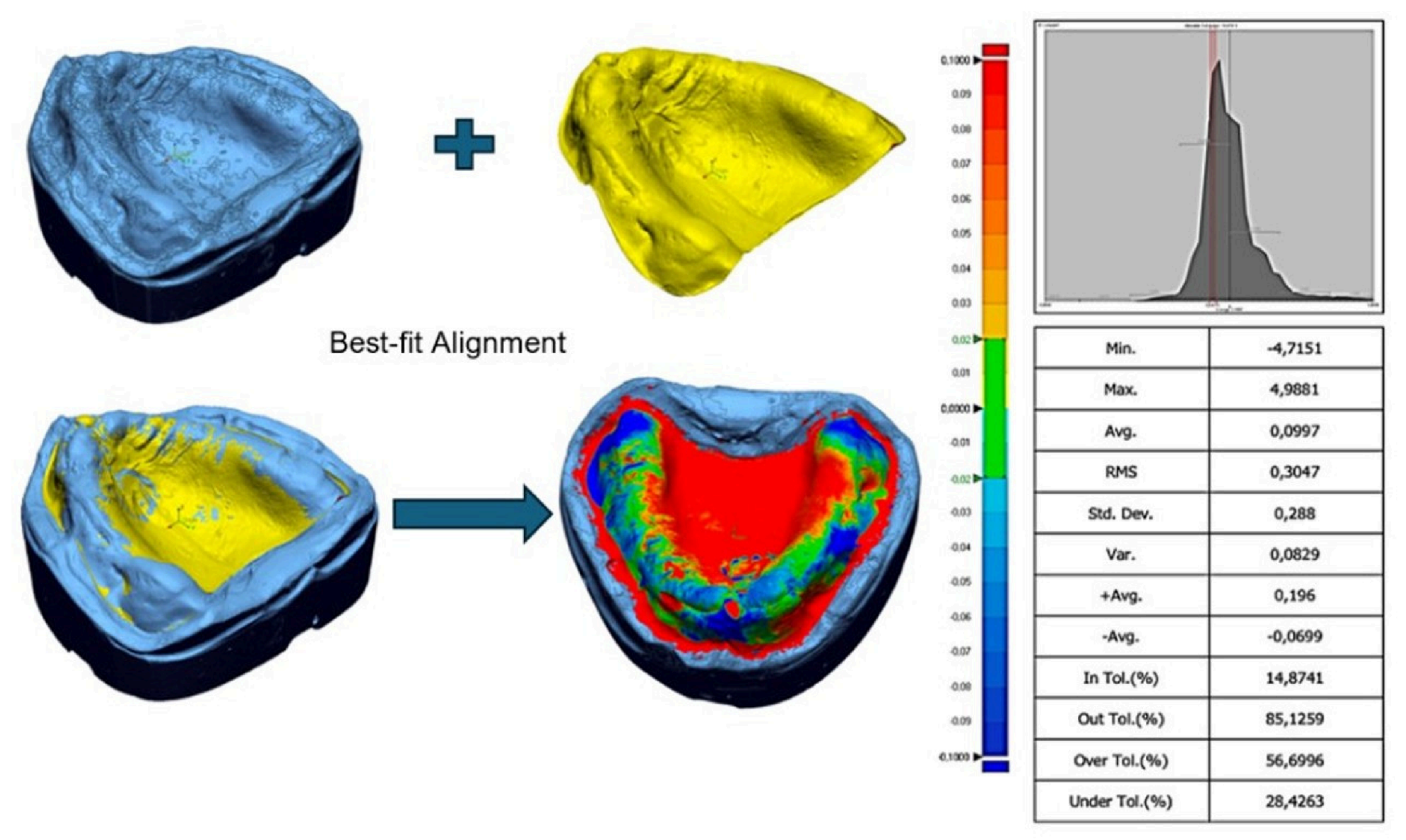
Schematic illustration of the denture base fit accuracy evaluation by best-fit superimposition and 3D deviation analysis.

Fit accuracy was evaluated by an examiner blinded to the fabrication workflow, and participants were unaware of their group allocation. All CDBs were fabricated only for research evaluation and were not intended for clinical delivery. To optimize scanning quality and minimize reflection, the intaglio surfaces of the denture bases were uniformly coated with a thin layer (<20 μm) of anti-glare scanning spray (Helling 3D Scan Spray; Helling GmbH, Heidgraben, Germany) according to the manufacturer’s instructions. All CDBs were scanned at high resolution using a calibrated industrial scanner (Solutionix C500, Solutionix Inc., Seoul, Republic of Korea), and the resulting datasets were archived in STL format on a per-patient basis using anonymized file codes. Digitized scans of the corresponding master casts were included to create complete datasets for all four fabrication workflows. All STL datasets were imported into surface-matching software (Geomagic Control X 2022, 3D Systems Corp., Rock Hill, SC, USA) for evaluation of fit accuracy. For each patient, the scanned master cast served as the reference dataset. Twenty-two predefined anatomical landmarks were identified on the master cast and used as standardized reference points for all measurements. The STL dataset of each corresponding denture base was then superimposed onto the reference master cast using the software’s best-fit alignment algorithm (Figure 3). Following optimal alignment, the mean 3D distance between the intaglio surface of each denture base and the corresponding master cast was measured at the predefined anatomical landmark locations.

The predefined anatomical landmarks were systematically distributed across six anatomical regions to provide representative coverage of anatomically and functionally relevant areas of the maxilla. Their distribution is summarized in Table 1, and their anatomical locations are illustrated in Figure 3. Superimposition and measurement procedures were performed by a trained examiner experienced in digital analysis and statistical evaluation.

**Figure 4:**
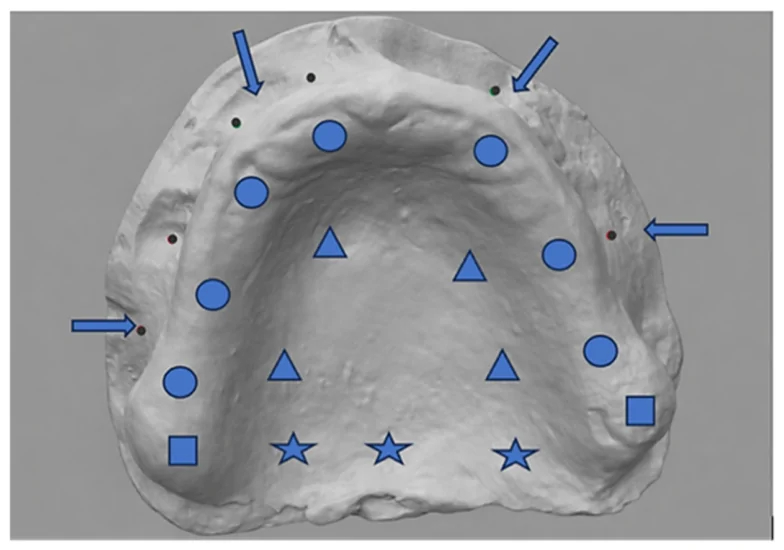
Anatomical locations and distribution of the predefined anatomical landmarks used to evaluate maxillary denture base fit accuracy. Circles represent the alveolar ridge crest, triangles the palatal region, squares the tuberosities, stars the posterior palatal seal, and arrows indicate the predefined anatomical landmarks located in the anterior and lateral border seal regions.

Alveolar ridge crest points were positioned along the mid-crestal line to evaluate fit within the primary support area. Palatal points were placed in the central palatal region to assess broad tissue contact. Anterior and lateral border seal points were selected from functional peripheral zones corresponding to denture border extension. Posterior palatal seal points were located along the vibrating line region. Tuberosity points were positioned bilaterally on the maxillary tuberosities to evaluate fit in posterior support areas (Figure 4).

This methodology allowed for deviations to be assessed not only overall but also regionally, thereby enabling a more detailed evaluation of fit accuracy. Color maps generated during the surface matching process were used to visualize the fit of the denture bases relative to the corresponding master casts. For color map visualization, a critical deviation threshold of ±0.02 mm was applied.

A broader tolerance of ±0.10 mm was used exclusively for numerical deviation analysis. These thresholds were selected based on previous digital accuracy studies to enhance visual sensitivity and enable standardized geometric comparison between workflows, rather than to define clinical acceptability.

### 2.6. Statistical Analysis

Statistical analyses were conducted utilizing IBM SPSS Statistics software (version 20.0; IBM Corp., Armonk, NY, USA). Descriptive statistics are presented as mean ± standard deviation and 95% confidence intervals. Denture bases fabricated for each participant using four distinct clinical workflows were evaluated. Deviation values derived from predefined measurement points were used to compare the workflows statistically. The Shapiro–Wilk test was used to assess the normality of the data distribution. In instances of normally distributed data, intergroup comparisons were performed using one-way analysis of variance (ANOVA), followed by Tukey’s post hoc multiple comparison test. Where data failed to meet the assumption of normality, the Kruskal–Wallis H test was applied, followed by pairwise comparisons with the Dunn–Bonferroni post hoc procedure. The threshold for statistical significance was established at *p* < 0.05, with *p* < 0.001 considered indicative of high statistical significance.

## 3. Results

### 3.1. Overall Fit Accuracy

Table 2 provides descriptive statistics and the results of pairwise comparisons for overall fit accuracy. Group C exhibited the lowest mean deviation (0.020 ± 0.073 mm), while the FD group presented the highest mean deviation (0.256 ± 0.607 mm). Pairwise comparisons revealed no statistically significant differences between the C and HE groups or between the FD and HL groups (*p* > 0.05). Conversely, all other pairwise comparisons demonstrated statistically significant differences (*p* < 0.001).

### 3.2. Region-Specific Fit Accuracy

Table 3 presents region-specific mean ± SD values with associated 95% confidence intervals, while Table 4 details pairwise comparisons.

In the tuberosity region, the C group exhibited the lowest mean deviation (0.099 ± 0.123 mm), and the FD group demonstrated the highest (0.380 ± 0.254 mm). Statistically significant differences were observed between the C–FD and C–HL groups (*p* < 0.001).

At the alveolar ridge crest, the C group again presented the lowest mean deviation (0.074 ± 0.109 mm), while the FD group showed the highest (0.316 ± 0.214 mm). No statistically significant difference was found between the C and HE groups (*p* = 0.362).

In the palatal region, statistically significant differences were only detected between the FD and HL groups, and the HE and HL groups (*p* < 0.05). The FD group exhibited the highest mean deviation (0.379 ± 0.244 mm), and the HL group presented the lowest (0.225 ± 0.165 mm).

At the anterior and lateral border seal regions, no statistically significant differences were detected between the C–HE and FD–HL groups (*p* > 0.05); however, all other pairwise comparisons revealed statistically significant differences (*p* < 0.001).

**Figure 5:**
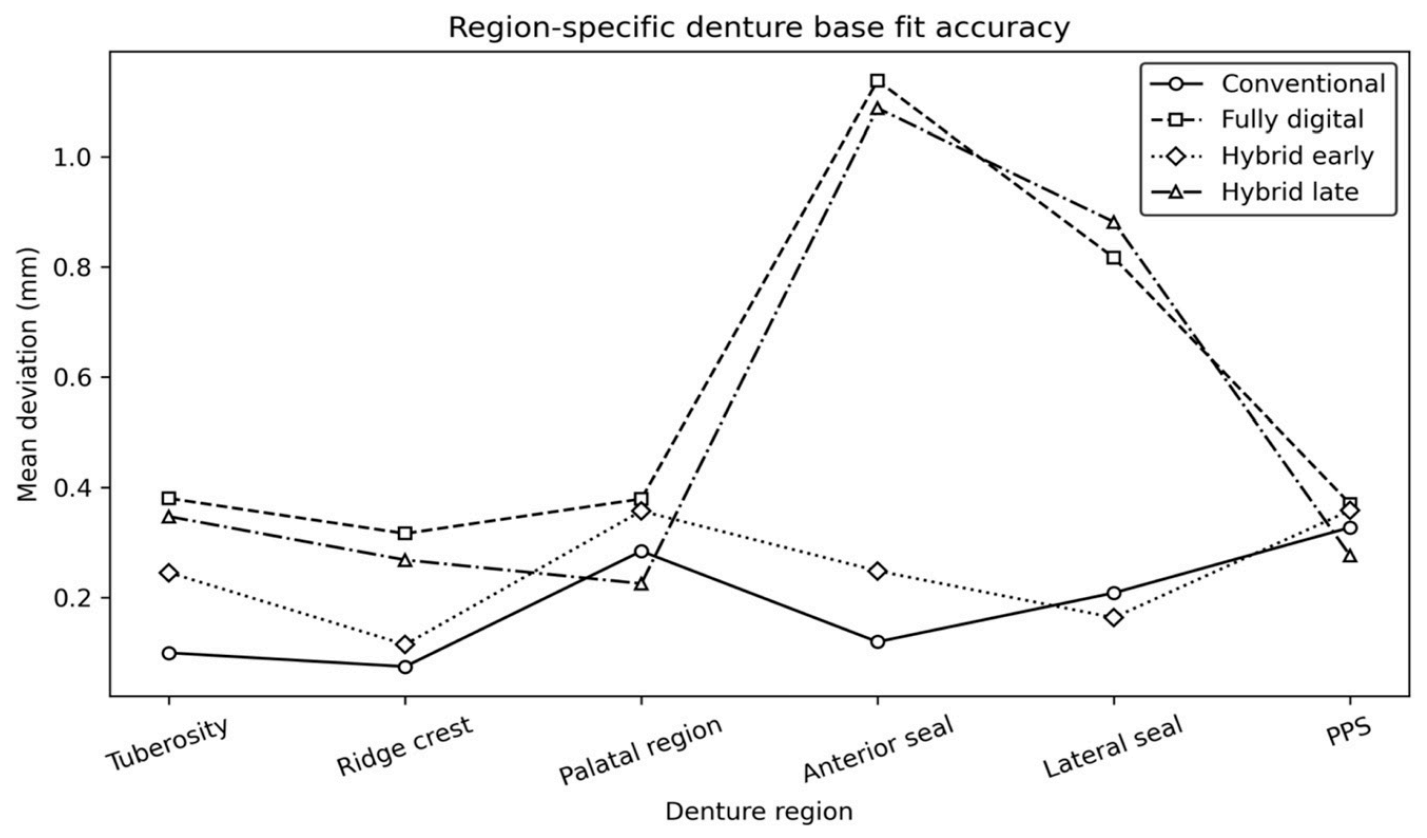
Region-specific mean denture base fit deviation values for each fabrication workflow. Mean deviation (mm) at predefined anatomical regions (tuberosity, alveolar ridge crest, palatal region, anterior border seal, lateral border seal, and posterior palatal seal) for the conventional, fully digital, hybrid early, and hybrid late workflows.

For the posterior palatal seal region, no statistically significant differences were observed among the groups (*p* = 0.287). Region-specific mean deviation values for denture base fit accuracy are graphically represented in Figure 5.

### 3.3. Color-Coded Deviation Maps

**Figure 6:**
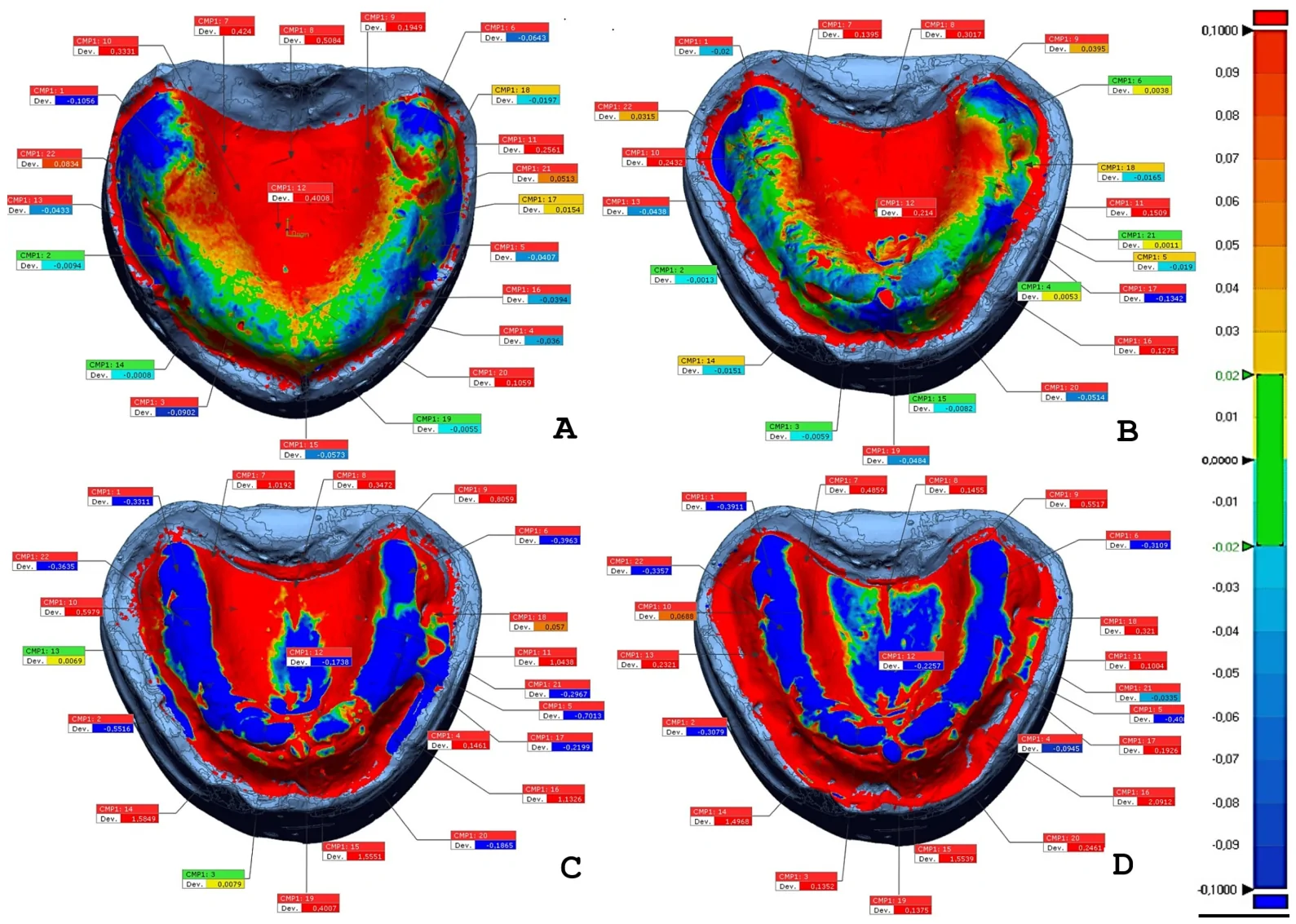
Color-coded deviation maps illustrating the fit accuracy of maxillary complete denture bases relative to the corresponding master casts for each fabrication workflow. (**A**) Conventional, (**B**) hybrid early, (**C**) fully digital, and (**D**) hybrid late. Green indicates deviations within the tolerance range (±0.02 mm), blue indicates negative deviations, and yellow-to-red indicates positive deviations.

Color-coded deviation maps (Figure 6) revealed that conventional and hybrid early workflows exhibited greater areas within tolerance, signifying better denture base adaptation. Conversely, fully digital and hybrid late workflows showed broader positive and negative deviations, especially in border seal and peripheral regions. These color patterns aligned with quantitative region-specific deviation measurements.

## 4. Discussion

Fully digital and hybrid approaches are increasingly incorporated into CD fabrication and may influence critical prosthetic parameters such as denture base fit and retention. Fit accuracy of CDBs is a key factor in mucosal adaptation and overall prosthesis performance [7]. This study evaluated the fit accuracy of CAD/CAM-fabricated complete denture bases (CDBs) using four different workflows. The first null hypothesis was partially rejected, with significant differences in overall fit accuracy found between some workflows but not others. Region-specific analysis revealed significant differences in fit accuracy across most anatomical regions, except for the posterior palatal seal area, where no significant differences were observed. Overall and region-specific analyses demonstrated that the C and HE groups achieved higher fit accuracy than the FD and HL groups. These findings may be related to the continued importance of functional impression procedures, as well as to current limitations associated with IOS and 3D-printing technologies.

Previous studies have revealed that although intraoral scanners offer notable advantages, direct intraoral scanning of edentulous arches remains inadequate for complete denture fabrication [2]. This limitation is primarily related to the mucostatic nature of IOS, which has been associated with lower trueness and border inaccuracies compared with immunocompressive conventional impression techniques, particularly in dynamic areas [13,14]. Al-Hamad et al. [15] classified edentulous arches into static and dynamic regions and reported that IOS demonstrated statistically significant differences compared with cast digitization, indicating lower trueness, although it may still be clinically feasible in areas of attached mucosa. In the present study, greater deviations were detected in the anterior and lateral border regions, particularly in the scan-derived denture bases, namely the FD and the HL groups. These regions are functionally dynamic and may be more prone to scanning inaccuracies due to tissue mobility and the presence of saliva [23].

Analysis showed that the lowest deviations were observed at the alveolar ridge crest, palatal, and tuberosity regions, in line with the previous literature [16,18]. Deviation values in the posterior palatal seal area were slightly higher than those in static regions, such as the tuberosity and alveolar ridge crest. Despite the posterior palatal seal’s importance for denture retention, this study found no significant differences between groups in that area, a finding consistent with reports suggesting digital approaches perform similarly to conventional methods [12,24]. Complete denture retention relies on accurate tissue adaptation, an effective peripheral seal, and adequate salivary adhesion. Denture base deformation, affected by shape, thickness, material, and processing, hinders mucosal adaptation, compromising retention, stability, and support [25]. Despite PMMA dentures’ susceptibility to deformation, the present study found comparable adaptation in critical retention areas (tuberosities, palate, posterior palatal seal) between heat-polymerized and 3D-printed dentures, suggesting that 3D-printed dentures may represent a viable alternative. While 3D-printing-technique-related deviations (support structures, build angle) influence geometric accuracy [26], mucosal adaptability allows dentures to tolerate minor soft tissue displacement. Controlled compression enhances retention, but excessive compression leads to inflammation and decreased retention. In contrast, greater misfit of the denture base intaglio surface in the posterior palatal seal region has been reported in previous studies [10,18,27,28], which was attributed to differences in fabrication techniques, scanner performance, and resin material properties. Chebib et al. [16] reported inferior retention of 3D-printed denture bases compared with conventional bases, potentially due to inaccurate determination of the finishing line on intraoral scans, leading to underextension or overextension of the denture borders. This finding is also relevant to denture base fit, as border accuracy plays a critical role in overall dimensional accuracy. Prosthesis retention, despite the absence of functional movement data, may have been maintained by the intraoral scanner’s close mucosal contact at rest [29,30]. Consistent mucostatic impressions from intraoral scanners could indicate that a complete peripheral seal is less critical. However, even if the border sealing is not perfect, denture treatment outcomes are reported to be not poor if there is no severe alveolar bone resorption or if overall excellent impression taking and occlusal relationship setting are achieved [31]. Kang et al. [2] found no significant performance differences between directly printed and conventionally fabricated complete dentures. They attributed this to the direct digitization process, which allows for improved denture base adaptation to the relaxed oral mucosa, potentially reducing the reliance on a strong peripheral seal when employing true mucostatic impression techniques [30,32]. Nonetheless, further investigation is warranted to comprehensively assess the impact of the peripheral seal on overall denture retention.

Recent systematic reviews suggest that digitally fabricated CDs provide clinical outcomes comparable to those of conventional dentures [33,34]. Studies on denture retention are mixed: Emera et al. [35] and Heikal et al. [36] found no significant difference between 3D-printed and conventional dentures (*p* > 0.05), while Maniewicz et al. [28] and Kortam et al. [37] reported superior retention for digitally fabricated dentures (*p* ≤ 0.05 and *p* < 0.001, respectively). Although Kortam et al. [37] and Heikal et al. [36] noted improved retention in all groups after three months (*p* < 0.001), Emera et al. [35] found no significant change over time (*p* > 0.05). Similarly, the present study demonstrated that the HL group CDBs exhibited lower deviations than those of the FD group at the alveolar ridge crest and palate, with palatal deviations comparable to those of the conventional group. Taken together, these findings suggest that denture retention is influenced by multiple factors beyond fabrication technique alone, warranting further investigation into the contribution of peripheral seal accuracy to overall prosthesis performance.

3D-printing parameters may also influence the fit accuracy of denture bases. Build orientation influences the internal fit of 3D-printed denture bases. A 45-degree build angle has been shown to improve manufacturing accuracy and internal fit [38]. Because the same build orientation was used in the present study, it may have contributed to the favorable fit accuracy observed for the printed denture bases [28,32,38].

As a result of the limitations of IOS, digitization of edentulous arches has predominantly been investigated using digitized impressions or casts (prefabricated or definitive), and hybrid scanning protocols have been recommended to provide more reliable outcomes [7,16,17,19]. Steinmassl et al. [18] used prefabricated casts and reported that impression-taking procedures for edentulous arches have no gold standard and are prone to variability. Variability may arise from factors such as the impression technique and the jaw’s anatomical features. Consequently, in the present study, both fully digital and hybrid workflows were evaluated, allowing for a comparison to be made between direct intraoral scanning of the edentulous arch and laboratory digitization of the master cast. The HL group demonstrated consistently lower deviation values than the FD group, suggesting that digitization of the definitive cast may reduce inaccuracies associated with direct intraoral scanning of edentulous arches, thereby providing a more reliable digital reference for denture fabrication.

Conventional fabrication of CDs has long been considered the standard technique because the denture base is fabricated on the definitive cast obtained from a functional impression. In the present study, the HE group was designed to combine conventional impression procedures with digital design and manufacturing. In an in vivo study, Maniewicz et al. [28] reported that the fit of conventionally fabricated CDBs was slightly superior to that of CAD-CAM denture bases, attributing this difference to the direct fabrication on the definitive cast. The findings of the present study are consistent with these results, as the C and HE group CDBs, in which denture bases were fabricated on definitive casts obtained from functional impressions, demonstrated better fit accuracy than the 3D-printed denture bases.

Limitations of conventional denture fabrication, such as polymerization shrinkage, have been addressed with the introduction of CAD/CAM systems [5,10,27]. However, polymerization shrinkage may still occur during the 3D-printing process because the material is not fully polymerized before the final post-processing stage. Additionally, deformation of 3D-printed CDBs may occur during removal from the build platform or during the alcohol-washing and post-curing phases [19]. Both 3D-printing and conventional fabrication techniques are affected by polymerization shrinkage, which may result in denture base misfit, as reported by Steinmassl et al. [18]. Furthermore, resin type and its compatibility with the 3D printer represent additional factors that may influence fit accuracy and should therefore be considered when interpreting the present findings.

In addition to fit accuracy, denture base adaptation plays a crucial role in evaluating CDs and has been investigated in the literature. Hsu et al. [7] evaluated denture base adaptation by measuring silicone thickness between the denture bases and the corresponding casts in an in vitro study. Conventional CDBs showed lower silicone thickness values than 3D-printed bases, indicating better adaptation. Similarly, the present study demonstrated superior fit accuracy for conventionally fabricated CDBs compared with 3D-printed dentures, further supporting the influence of fabrication workflow on denture base adaptation.

Although the present study demonstrated statistically significant differences in denture base fit accuracy among the evaluated workflows, the clinical implications of these findings should be considered with caution. Improved denture base adaptation may contribute to better retention and stability; however, clinical performance is influenced by multiple biological and prosthetic factors beyond fit accuracy alone. Therefore, further clinical studies incorporating patient-reported outcomes and objective retention assessments are needed to determine whether the observed differences translate into clinically meaningful benefits.

The findings of the present study should be interpreted while keeping its limitations in mind. Although the sample size fulfilled the minimum requirement determined by an a priori power analysis, the relatively small number of participants remains an important limitation and may reduce the generalizability of the findings. The exclusion of a subtractive manufacturing workflow represents another limitation of this study, as comparisons could only be made between conventional and additively manufactured denture bases. Furthermore, the present study focused exclusively on the geometric fit accuracy of denture bases and did not include patient-reported outcome measures or objective clinical assessments of denture retention, stability, masticatory performance, or patient comfort. Therefore, the clinical significance of the observed differences in fit accuracy could not be directly established. The study also evaluated only a single 3D-printing resin, without considering the potential influence of different material properties, printing parameters, and post-processing protocols. Future multicenter studies with larger patient cohorts should explore long-term clinical outcomes, patient-reported outcomes, variations in printing parameters, and comparisons across different digital manufacturing systems. Finally, intaglio surface adaptation was evaluated extraorally, without simulating the dynamic oral environment, the presence of saliva, or variations in ridge morphology, which should be considered in future studies.

## 5. Conclusions

Workflow selection significantly impacts the accuracy of denture base fit. Conventional and hybrid early workflows provided the highest fit accuracy, whereas fully digital and hybrid late workflows exhibited greater deviations. The hybrid early approach demonstrated fit accuracy comparable to the conventional workflow, whereas the hybrid late approach showed a pattern similar to the fully digital workflow. Further research should incorporate longitudinal clinical outcomes, patient-reported outcomes, objective retention assessments, and comparative analyses of different digital manufacturing techniques to refine digital denture fabrication.

## Figures and Tables

**Table 1:** Distribution of predefined anatomical landmarks in the maxillary arch (*n* = 22).

Regions	Point Distribution ^1^
Alveolar ridge crest	7
Palate	4
Anterior border seal	3
Lateral border seal	3
Posterior palatal seal	3
Left–right tuberosity	2

^1^ Points indicate predefined anatomical measurement locations used for denture base fit deviation analysis. *n*, total number of measurement points.

**Table 2:** Overall fit accuracy values (mean ± SD, 95% CI) for the study groups.

Groups	Mean ± SD (95% CI) (mm)
C	0.020 ± 0.073 (0.011–0.028) ^a^
FD	0.256 ± 0.607 (0.186–0.327) ^b^
HE	0.031 ± 0.101 (0.019–0.043) ^a^
HL	0.243 ± 0.572 (0.176–0.309) ^b^

Kruskal—Wallis test followed by Dunn–Bonferroni post hoc test. Different superscript letters indicate statistically significant differences (*p* < 0.05). C = conventional; FD = fully digital; HE = hybrid early; HL = hybrid late. Different lowercase letters indicate statistically significant differences among groups (*p* < 0.05).

**Table 3:** Region-specific denture base fit accuracy values (mean ± SD, 95% CI) (mm).

Region	C (Mean ± SD, 95% CI; mm)	FD (Mean ± SD, 95% CI; mm)	HE (Mean ± SD, 95% CI; mm)	HL (Mean ± SD, 95% CI; mm)
Tuberosity	0.099 ± 0.123 (0.049–0.149)	0.380 ± 0.254 (0.277–0.483)	0.245 ± 0.195 (0.166–0.324)	0.347 ± 0.133 (0.293–0.401)
Alveolar ridge crest	0.074 ± 0.109 (0.051–0.097)	0.316 ± 0.214 (0.271–0.360)	0.114 ± 0.115 (0.090–0.138)	0.268 ± 0.190 (0.229–0.307)
Palatal region	0.284 ± 0.219 (0.221–0.344)	0.379 ± 0.244 (0.311–0.447)	0.357 ± 0.237 (0.291–0.423)	0.225 ± 0.165 (0.179–0.271)
Anterior border seal	0.119 ± 0.139 (0.074–0.165)	1.138 ± 0.863 (0.853–1.421)	0.248 ± 0.281 (0.157–0.340)	1.088 ± 0.881 (0.802–1.374)
Lateral border seal	0.208 ± 0.549 (0.033–0.384)	0.817 ± 0.677 (0.594–1.039)	0.163 ± 0.183 (0.103–0.222)	0.882 ± 0.608 (0.685–1.079)
Posterior palatal seal (PPS)	0.327 ± 0.216 (0.257–0.397)	0.370 ± 0.244 (0.291–0.449)	0.358 ± 0.263 (0.273–0.444)	0.276 ± 0.195 (0.213–0.340)

Values are presented as mean ± standard deviation with 95% confidence intervals. C = conventional; FD = fully digital; HE = hybrid early; HL = hybrid late.

**Table 4:** Region-specific pairwise comparisons between the groups.

Region	Comparison	*p* Value
Left–right tuberosity	C vs. FD	<0.001 *
	C vs. HE	0.056
	C vs. HL	<0.001 *
	FD vs. HE	0.057
	FD vs. HL	0.915
	HE vs. HL	0.201
Alveolar ridge crest	C vs. FD	<0.001 *
	C vs. HE	0.362
	C vs. HL	<0.001 *
	FD vs. HE	<0.001 *
	FD vs. HL	<0.001 *
	HE vs. HL	<0.001 *
Palate	C vs. FD	0.197
	C vs. HE	0.458
	C vs. HL	0.577
	FD vs. HE	0.998
	FD vs. HL	0.002 *
	HE vs. HL	0.008 *
Anterior border seal	C vs. FD	<0.001 *
	C vs. HE	0.812
	C vs. HL	<0.001 *
	FD vs. HE	<0.001 *
	FD vs. HL	0.987
	HE vs. HL	<0.001 *
Lateral border seal	C vs. FD	<0.001 *
	C vs. HE	0.997
	C vs. HL	<0.001 *
	FD vs. HE	<0.001 *
	FD vs. HL	0.997
	HE vs. HL	<0.001 *
Posterior palatal seal	C vs. FD	0.845
	C vs. HE	0.931
	C vs. HL	0.772
	FD vs. HE	0.996
	FD vs. HL	0.287
	HE vs. HL	0.402

One-way ANOVA or Kruskal–Wallis test followed by appropriate post hoc multiple comparisons. * *p* < 0.05 indicates statistically significant difference.

## Data Availability

The original contributions presented in this study are included in the article. Further inquiries can be directed to the corresponding author.

## Abbreviations

CDBs	Complete denture bases
CDs	Complete dentures
C	Conventional
FD	Full digital
HE	Hybrid early
HL	Hybrid late
PMMA	Polymethyl methacrylate
IOS	Intraoral scanning
CAD	Computer-aided design
3D	3-dimensional
